# Long-term effects of cranial irradiation and intrathecal chemotherapy in treatment of childhood leukemia: a MEG study of power spectrum and correlated cognitive dysfunction

**DOI:** 10.1186/1471-2377-12-84

**Published:** 2012-08-28

**Authors:** Marita Daams, Ilse Schuitema, Bob W van Dijk, Eline van Dulmen-den Broeder, Anjo JP Veerman, Cor van den Bos, Leo MJ de Sonneville

**Affiliations:** 1 Department of Radiology, VU University Medical Center, Amsterdam, The Netherlands; 2 Department of Clinical Child and Adolescent Studies, Faculty of Social Sciences & Leiden Institute for Brain and Cognition, Leiden University, Wassenaarseweg 52, P.O. Box 9555, 2300 RB, Leiden, The Netherlands; 3 Department of Pediatrics, Division of Pediatric Hematology/Oncology, VU University Medical Center, Amsterdam, The Netherlands; 4 Department of Physics and Medical Technology and the department of Clinical Neurophysiology, VU University Medical Center, Amsterdam, The Netherlands; 5 Department of Pediatric Oncology, Emma Children’s Hospital, Amsterdam Medical Center, Amsterdam, The Netherlands

**Keywords:** Late effects, Childhood cancer, Magnetoencephalography, Resting state, Oscillatory power, Neuropsychology, Accelerated aging

## Abstract

**Background:**

Prophylaxis to prevent relapses in the central nervous system after childhood acute lymphoblastic leukemia (ALL) used to consist of both intrathecal chemotherapy (CT) and cranial irradiation (CRT). CRT was mostly abolished in the eighties because of its neurotoxicity, and replaced with more intensive intrathecal CT. In this study, a group of survivors treated with CRT before 1983 and another group treated without CRT thereafter are investigated 20–25 years later, giving a much stronger perspective on long-term quality of life than previous studies. The outcomes will help to better understand these groups’ current needs and will aid in anticipating late effects of prophylactic CRT that is currently applied for other diseases. This study evaluates oscillatory neuronal activity in these long-term survivors. Power spectrum deviations are hypothesized to correlate with cognitive dysfunction.

**Methods:**

Resting state eyes-closed magnetoencephalography (MEG) recordings were obtained from 14 ALL survivors treated with CT + CRT, 18 treated with CT alone and 35 controls. Relative spectral power was calculated in the δ, θ, α1, α2, β and γ frequency bands. The Amsterdam Neuropsychological Tasks (ANT) program was used to assess cognition in the executive functions domain. MEG data and ANT scores were correlated.

**Results:**

In the CT + CRT group, relative θ power was slightly increased (p = 0.069) and α2 power was significantly decreased (p = 0.006). The CT + CRT group performed worse on various cognitive tests. A deficiency in visuomotor accuracy, especially of the right hand, could be clearly associated with the deviating regional θ and α2 powers (0.471 < r < 0.697). A significant association between decreased regional α2 power and less attentional fluctuations was found for CT + CRT patients as well as controls (0.078 < r < 0.666). Patients treated with CT alone displayed a power spectrum similar to controls, except for a significantly increased level of left frontal α2 power (p = 0.030).

**Conclusions:**

The tendency towards global slowing of brain oscillatory activity, together with the fact that dementia has been reported as a late effect of CRT and the neuropsychological deficiencies currently present, suggest that the irradiated brain might be aging faster and could be at risk for early‐onset dementia. The CT group showed no signs of early aging.

## Background

Acute lymphoblastic leukemia (ALL) is the most common malignancy diagnosed in children, representing almost one third of all childhood cancers. There is a peak incidence at 2–5 years of age. Over the last decades, cure rates for ALL patients have gone from less than 5% to over 80% due to improved treatment protocols [1]. An essential part of ALL treatment is central nervous system (CNS) prophylaxis, mostly performed by cranial irradiation (CRT) and/or intrathecal chemotherapy (CT). In the Netherlands, CRT was abolished in 1983 [2].

As survival rates for ALL continue to rise, the focus of clinicians is increasingly shifting to the late effects of therapy and related quality of life [3]. CRT patients have a 13.6 times higher chance of developing secondary neoplasms in the irradiated field within 20 years following treatment [4]. Late neurotoxic effects of CRT are also well described. Many studies have reported a causative link between prophylactic CRT and long-term neurocognitive deficits among ALL survivors, for example memory problems, cognitive slowing and attention deficits [5-8]. Cognitive effects beyond 10 years after treatment are largely unknown [9]. This study provides data from patients, on average 25 years post childhood treatment, giving a much stronger perspective on long-term quality of life than previous studies. These outcomes not only help to better understand these groups’ current needs, but can also aid in anticipating late effects of currently applied prophylactic CRT that is still part of treatment for ALL in some countries, but also for brain tumours and small cell lung cancer.

Neurocognitive late effects of chemotherapy alone are relatively subtle compared to the effects of CRT. However, a significant percentage of children that received chemotherapy alone also suffer from neurocognitive deficits, particularly in the domain of executive functioning (EF) [10-13]. The underlying mechanisms of neurotoxicity, caused by either CRT or CT, are not yet fully understood. Structural imaging has revealed smaller white matter volumes in this population, which correlated with cognitive dysfunction [9]. Functional neuroimaging methods like functional magnetic resonance imaging (fMRI), electroencephalography (EEG) or magnetoencephalography (MEG) have rarely been applied in late effect studies of cancer treatment, while these methods are very suitable to study subtle brain dysfunction. Among the available functional imaging techniques, MEG has the unique property of combining high temporal resolution with good spatial resolution. The MEG signal is quantified in spectral power, which indicates the strength of magnetic induction fields generated by oscillatory neuronal activity and is decomposed into frequency bands [14]. Relative power is the percentage of power in any band compared with the sum of the power of all frequency bands. A power spectrum that displays a shift towards relatively more power in the lower frequency bands is generally considered to be a pathological sign of slowing brain activity and is associated with deteriorated cognition [15,16]. Also, increased δ or θ power is known as a nonspecific sign of brain pathology [17]. In this paper we will describe relative spectral power changes assessed by MEG during resting state with eyes closed. To our knowledge, this is the first study to use MEG in this population. We hypothesize that the pattern of oscillatory activity will deviate from that of controls and that the deviations will correlate with cognitive dysfunction.

In 1989, Tucker et al. conducted a study of long-term neurocognitive effects (11.5 ± 7 years after treatment) of CT + CRT in ALL and Non Hodgkin Lymphoma (NHL) survivors treated at age 15–69 [18]. They compared the effects between a group treated at 25 years of age or younger and a group treated at an older age. In the younger group, abnormalities were more outspoken, indicating higher susceptibility to neurotoxicity at a younger age. Using EEG, they found excess θ activity and slightly slowed α. The abnormalities were interpreted as minor, but relatively more severe in the younger group, reflecting subtle cerebral cortical dysfunction. Therefore, an association between worse outcome and younger age at treatment is hypothesized.

## Methods

### Subjects

Seventy‐five eligible ALL survivors were identified from patient records of the VU University Medical Center (VUMC) and the Academic Medical Center (AMC) Amsterdam. They were sent a letter explaining the study and were subsequently contacted by phone. Unfortunately, 45% were unwilling to participate or met one or more of the exclusion criteria (i.e. use of centrally acting drugs, active psychiatric disease or symptoms, pre-existing CNS disorders, metal parts in the body or pregnancy). Survivors who were willing to participate were requested to recruit a control (sibling, partner or friend, n = 44). The ALL survivors were treated according to two different protocols developed by the Dutch Childhood Leukemia Study Group (DCLSG). The first group, CT + CRT (n = 18) was treated according to DCLSG protocol ALL-5 (1979–1984), which consisted of standard dose CT (intravenously administered vincristine, prednisone, L-asparaginase, daunorubicine, intrathecal injections of methotrexate (MTX) and prednisolone) and CRT (2500 Gy). The second group, CT (n = 23), was treated according to DCLSG protocol ALL-6 (1984–1988), which consisted of standard dose CT (intravenous vincristine, dexamethasone, L-asparaginase, intrathecal MTX and prednisolone), without CRT. Duration of treatment was approximately 2 years.

Two CT + CRT patients were excluded from data analysis because meningiomas were discovered during assessment. Additionally, data from two CT + CRT patients, five CT patients and nine controls could not be used due to artefacts discussed in the magnetoencephalography section below. None of the CT patients or controls presented with CNS disorders. Eventually, data from 14 CT + CRT patients, 18 CT patients and 35 controls were used in the analysis for this paper. Characteristics of these participants are reported in Table 1.

**Table 1:** Characteristics of the included subjects

	**CT + CRT (n = 14)**	**CT (n = 18)**	**Controls (n = 35)**
Gender, male % (n)	57.1 (8)	44.4 (8)	45.7 (16)
Age at assessment, y, *M (SD)*	31.0 (4.3)	24 (2.9)	26.6 (6.1)
Age at diagnosis, y, *M (SD)*	5.7 (3.4)	4.1 (2.0)	N/A
Time since diagnosis, y, *M (SD)*	25.3 (2.5)	20.0 (2.0)	N/A
Estimated IQ, *M (SD)*	91.9 (18.1)	105.8 (19.9)	108.8 (19.4)

IQ was estimated using a 4-subtest short-form of the Wechsler Adult Intelligence Scale Revised (WAIS-R III). *M* mean; *SD* standard deviation.

The ethical principles of the Helsinki Declaration were followed and approval was obtained from the Medical Ethics Committee of the VU University Medical Center (n° 2006/200). All participants signed an informed consent form.

### Magnetoencephalography

MEG was recorded for ten minutes while subjects were inside a magnetically shielded room (Vacuumschmelze GmbH, Germany) using a 151 channel whole-head MEG system (CTF systems Inc., Canada) [19]. A third order software gradient was used with a recording passband of 0.25-125 Hz and a sample frequency of 625 Hz in 49 subjects (assessment around 2009), and a sample frequency of 312.5 Hz in 18 subjects (assessment around 2007) [20]. Both sample frequencies occurred in each of the subject groups. Head position was monitored. MEG was recorded during a no-task, eyes-closed resting state condition. For this study, 149 of the 151 channels could be used. Additionally, 7 channels appeared to produce no signal for some of the subjects and were appropriately excluded from the calculation of the mean global and regional powers per subject. MEG recordings were converted to ASCII files and from these files four artefact free epochs of 13 s per subject were carefully selected by visual inspection by the two first authors (based on consensus). The MEG data were filtered in the following frequency bands: δ (0.5-4 Hz), θ (4–8 Hz), α1 (8–10 Hz), α2 (10–12 Hz), β (13–30 Hz) and γ (30–50 Hz). Hypothetically, the lower and upper α bands are associated with different cognitive processes and many studies have found differential effects in these subbands [21,22]. There is no such rationale for subdividing the other frequency bands. Data in the δ band were largely corrupted by movement artefacts like breathing. Therefore, all subjects with artefacts in the δ band were excluded and group differences were investigated. Within this artefact‐free dataset (CT + CRT n = 10, CT n = 11, controls n = 12), the groups did not differ on δ power. Therefore, the δ band was disregarded and more subjects with artefact‐free data in the other frequency bands were included. Eventually, only 16 subjects (CT + CRT n = 2, CT n = 5, CON n = 9) were excluded due to movement artefacts in the other frequency bands, mostly caused by eye blinks and heart beats.

**Figure 1:**
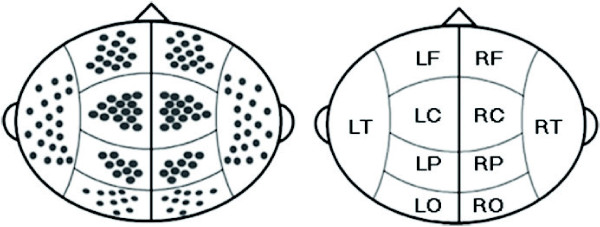
**Schematic projection of MEG channels.** Head drawing to illustrate the clustering of channels per region. Midline channels were disregarded. L = left, R = right, F = frontal, C = central, P = parietal, O = occipital, T = temporal.

Relative instead of absolute powers were analyzed, because absolute powers are partially determined by the distance between the MEG sensor and the measured neural substrate. This would result in misleading outcomes, because part of our population has a smaller head size due to CRT [23]. The data were processed using CTF DataEditor and BrainWave 0.8.80 [19,24]. Relative band powers were computed for four 13 s epochs for each subject in the five frequency bands, and averaged for each subject. MEG channels were clustered based upon the approximate underlying cortical areas (see Figure 1). Regional means were calculated averaging relative powers of the available channels per cluster, over four epochs. 

### Neurocognitive assessment

The Amsterdam Neuropsychological Tasks (ANT) program was used to assess EF [25]. The computerized ANT provides for highly standardized assessments and automated recordings of speed and accuracy of information processing, attention processes and working memory. It has proven to be helpful in defining neurocognitive deficit profiles in various clinical domains associated with a generally diffuse impact on the brain, such as phenylketonuria, multiple sclerosis, neurofibromatosis, and middle late effects of childhood ALL [12,13,26-31]. Based on these studies, outcome parameters of baseline response speed, complex visual information processing, sustained attention, work pace and attentional fluctuations, cognitive flexibility (set shifting and inhibition), visuomotor skills, and visuospatial sequential working memory were selected for assessment of this study’s population. Short task descriptions are given in Table 2, and in more detail in Additional file 1. The reliability and validity of these tasks have extensively been described elsewhere [32]. Task parameters that discriminated between the groups were selected for correlation with MEG data. 

**Table 2:** Amsterdam Neuropsychological Tasks program: description of subtests

**TASK**	**VARIABLE**	**LABEL**
*Baseline speed*	T_bs	Baseline speed (simple reaction time)
	S_bs	Baseline speed stability
*Feature Identification*	Ts_fi	Speed of processing complex visuospatial information (similar condition)
	Td_fi	Speed of processing simple visuospatial information (dissimilar condition)
	diffT_fi	*Ts_fi minus Td_fi*; Extra response time needed for higher order complex visuospatial information processing
*Memory Search Objects 2D*	T1_2d	Speed of working memory search processes (low memory load condition); Target detection
	T2_2d	Speed of working memory search processes (high memory load condition); Requires continuous monitoring and updating of the contents of the working memory
	diffT_2d	*T2_2d minus T1_2d*; Extra response time needed for memory search processes
*Sustained attention*	T_sa	*Work pace;* Response speed on sustained attention
	SD_sa	*Attentional fluctuations;* Response speed stability
	PM_sa	Percentage of misses (errors on target signals)
	PF_sa	Percentage of false alarms (errors on nontarget signals)
*Shifting attentional set*	T_inhib	Speed of *inhibition*
	P_inhib	Accuracy of *inhibition* (% errors)
	T_flex	Speed of *set shifting* (flexibility)
	P_flex	Accuracy of *set shifting* (flexibility)(% errors)
*Tracking*	Da_tr	*Visuomotor accuracy;* Mean absolute distance to ideal trajectory, mean left and right hand
	S_tr	*Visuomotor stability;* mean left and right hand
	Dal(r)_tr	*Visuomotor accuracy;* left (right) hand
*Pursuit*	D_pu	*Visuomotor accuracy;* Distance to randomly moving target, mean left and right hand
	S_pu	*Visuomotor stability*, mean left and right hand
	Dl(r)_pu	*Visuomotor accuracy;* left (right) hand
	Sl(r)_pu	*Visuomotor stability;* left (right) hand
*Visuospatial sequencing*	Nit_vs	*Visuospatial working memory;* Number of correctly identified dots in a visuospatial pattern
	Nitco_vs	*Sequential visuospatial working memory;* Number of correctly identified dots in correct order
	diff_Nit_Nitco	*Nit_vs minus Nitco_vs*; Accuracy of sequential working memory processes

For more elaborate descriptions of these subtests, see Additional file 1.

### Statistical analyses

Individuals’ global and regional means of relative power were entered in SPSS software (version 16.0; SPSS Inc, Chicago, IL) for statistical analyses. All powers were transformed according to the following logarithmic function [33]:

$$\text{power}\ln{=}^{e}\log\left(\frac{\text{power}}{1-\text{power}}\right)$$

Global relative power differences between subject groups were analyzed using a Multivariate General Linear Model (GLM) with group as between-subjects factor and the frequency bands as dependent variables. Age was used as a covariate when significantly correlated with the dependent variable within the healthy control group. Wilks’ Lambda corrected F and p-values are reported. For each frequency band, regional power differences between groups were analyzed using a MANOVA for ten regions (bilateral central, frontal, parietal, occipital and temporal power). Differences between groups were tested with simple contrasts comparing each patient group with controls. Partial eta squared (η_p_^2^) was computed to estimate effect sizes (weak effect: η_p_² ≈ 0.03; moderate: η_p_² ≈ 0.06; large: η_p_² ≥ 0.14) [34]. Cohen’s d was used for simple contrasts (small effect: 0.2 < d < 0.3; medium: 0.3 < d < 0.8; large: d > 0.8).

### Correlations between regional powers and discriminative cognitive variables

The ANT variables that discriminated between patients and controls were correlated with MEG regional power outcomes in the discriminating frequency bands. Pearson’s correlations (r) and their p-values were calculated between regional powers and these neuropsychological variables, controlling for age. When r in the patient group or in the control group was significant, the r-values were compared. First, they were transformed into z'-values according to the Fisher transformation [35]. The difference |z*| between r_1_ within the patient group and r_2_ within controls was calculated and significance of that difference was determined by comparing |z*| to normal z-values [35].

### Linear regression models of powers predicting cognitive performance

Separately for each frequency band, regional means of relative powers that differed significantly between groups were entered into linear regression analysis predicting cognitive parameters that discriminated between groups, together with age. The backward method was used, using a probability (F) of 0.05 for entry and 0.10 for removal.

## Results

### Global relative power

After ^e^log transformation, separate inspection per group revealed normal distributions within all frequency bands, except for α1 in controls (SW statistic: 0.926 (p = 0.022)). Based on these results, the ^e^log transformation was considered sufficiently effective to allow for parametric statistical testing.

**Figure 2:**
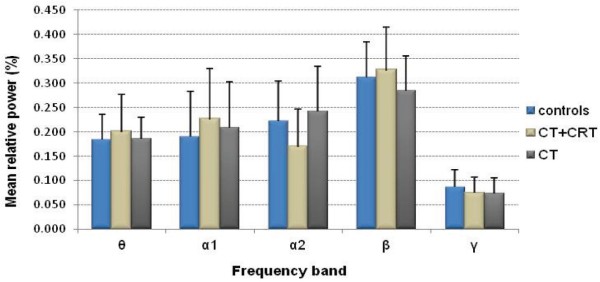
**Mean global relative power values.** X-axis: Frequency band; y-axis: Mean global relative power (%). Error bars represent one standard deviation.

The groups differed significantly in age (p = 0.001) due to the consecutive time periods the treatment protocols were applied. The correlation analyses within the control group between the powers and age resulted in a significant outcome for θ_ln (r = −0.560, p = 0.0005), so age was used as a covariate in the multivariate power analyses. The GLM results for the mean global relative powers are shown in Table 3. In the CT + CRT group, θ power was slightly increased compared to controls and α2 power was significantly decreased. Within the CT group, global power levels did not differ from controls. To facilitate interpretation, Figure 2 displays the *untransformed* mean global relative power values per group for all frequency bands. For additional graphs of transformed and untransformed global relative powers, see Additional file 2.

**Table 3:** Global relative power differences

***MANOVA***	**Between-Subjects effects**						**Simple contrasts (versus controls)**			
	**AGE**	**Sign. (p)**	**Effect size (η**_**p**_^**2**^**)**	**GROUP**	**Sign. (p)**	**Effect size (η**_**p**_^**2**^**)**	**CT + CRT group**		**CT group**	
							**Sign. (p)**	**Cohen’s d**	**Sign. (p)**	**Cohen’s d**
**Multivariate tests**	F[5,59] = 2.516	0.039	0.176	F[10,118] = 1.809	0.066*	0.133	N/A	N/A	N/A	N/A
θ_ln	F[1,63] = 12.404	0.001	0.164	F[2,63] = 2.191	0.120	0.065	0.069*	0.190	0.582	0.061
α1_ln	F[1,63] = 1.589	0.212	0.025	F[2,63] = 1.057	0.354	0.032	0.152	0.335	0.693	0.201
α2_ln	F[1,63] = 4.404	0.040	0.065	F[2,63] = 5.757	0.005**	0.155	0.006**	−0.719	0.243	0.207
β_ln	F[1,63] = 1.509	0.224	0.023	F[2,63] = 0.511	0.602	0.016	0.889	0.171	0.347	−0.371
γ_ln	F[1,63] = 1.810	0.183	0.028	F[2,63] = 1.684	0.194	0.051	0.117	−0.386	0.218	−0.448

Differences were tested between the CT + CRT group (n = 14), the CT group (n = 18) and controls (n = 35) using multivariate GLM, with age as a covariate. * = statistical trend, ** = significant difference.

### Regional relative power

**Figure 3:**
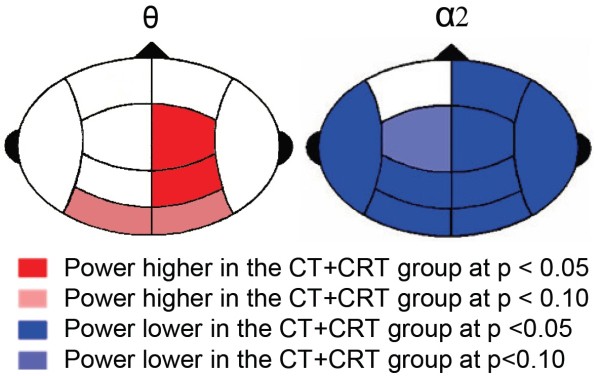
**Visualization of regional power differences between the CT + CRT group and controls.** Differences concern the θ and α2 frequency bands. The directions of the deviations are colour coded per region (red for increases, blue for decreases).

For each frequency band, a separate MANOVA was used to test for regional power differences between groups in the ten regions shown in Figure 1. The multivariate tests did not reach significance, with just a trend for the group difference on regional α2 powers (p = 0.067). Group contrasts versus controls within the θ band revealed increased powers for the CT + CRT group in the RC (p = 0.038) and RP (p = 0.029) regions and trends for increases in the LO (p = 0.066) and RO (p = 0.051) regions. This group also displayed two trends for increased levels of α1 power in the LO (p = 0.081) and RO (p = 0.059) regions. Within the α2 band, the CT + CRT group displayed decreased power levels in all regions, except for LF. The CT group only displayed a significant increase of α2 power (p = 0.030) in the left frontal region compared to controls. For a table of all statistics on the multivariate tests and contrasts, see Additional file 3. Significant contrast results for the CT + CRT group versus controls are displayed in Figure 3. The directions of the deviations are colour coded per region (red for increases, blue for decreases). No significant regional *Group x Hemisphere* interactions were found.

The time that has passed since treatment could hypothetically allow for either recovery or worsening of late effects. Therefore, time since diagnosis was correlated with powers in all frequency bands, separately within each patient group. There were no significant correlations with either global or regional powers. Hypothetically, a younger age could mean more vulnerability to neurotoxicity of treatment. However, no effect of age at diagnosis was found on either global or regional powers.

### Group differences on cognition

The cognitive variables are explained in Additional file 1. Age did not correlate significantly with any of the cognitive parameters within the control group and was therefore not used as a covariate. The CT + CRT group performed worse than controls on measures of cognitive flexibility (percentage of errors (P_flex, p = 0.002), attentional fluctuations during sustained attention (SD_sa, p = 0.062), visuomotor accuracy (D_pu, p = 0.049; S_pu, p = 0.075; Dr_pu, p = 0.015; Sr_pu, p = 0.029) and sequential visuospatial working memory (Nit_vs, p = 0.065; Nitco_vs, p = 0.023; Nit-Nitco_vs, p = 0.036). A table of statistics, including effect sizes, can be found in Additional file 4. When the CT group was compared with controls, trends were found for worse sequential visuospatial working memory (Nitco_vs, p = 0.092) and accuracy of inhibition (P_inhib, p = 0.059).

### Correlations between regional powers and discriminative cognitive variables

Correlations were calculated within the composite group of CT + CRT patients and controls to investigate general patterns. Significantly differentiating regional powers were correlated with differentiating cognitive variables. Increased levels of RC and RP θ power correlated significantly with worse performance on visuomotor control (D_pu, Dr_pu and Sr_pu). Decreased α2 powers in the eight significant regions of the CT + CRT group (LO, LP, LT, RC, RF, RO, RP and RT) were significantly correlated with better performance on attentional fluctuations (SD_sa) and with worse performance on visuomotor control (D_pu and Dr_pu). Correlations within the CT + CRT group and within controls were separately calculated and then compared (see Additional file 5). There was a trend for the difference between CT + CRT patients and controls in correlation coefficients of attentional fluctuations with right frontal α2 powers. This correlation was absent in the CT + CRT group (r = 0.078), while it was significantly positive in controls (r = 0.549). Correlations between visuomotor accuracy of the right hand (Dr_pu) and regional α2 powers differed significantly in seven regions (not in RF) between CT + CRT patients (where there were significantly negative correlations with r ranging from −0.508 to −0.697)), and controls (where the correlations were not significant and ranging from −0.129 to 0.142). Roughly the same pattern was seen in the θ band, but with positive correlations within the CT + CRT group between Dr_pu and RC θ (r = 0.630) and RP θ (r = 0.599).

To verify whether the increased LF α2 power level in the CT group was associated with the mild deficiencies in accuracy of inhibition and sequential visuospatial working memory, these outcomes were correlated, but no significant results were found. The significant correlations between α2 and attentional fluctuations observed in controls were absent in the CT group.

To explore the possibility that increased levels of θ power reflect the results of compensatory activity, the data were searched for associations between increased regional θ powers and *better* cognitive performance on any of the neuropsychological tasks. Two trends were found for increased left occipital θ power in association with less attentional fluctuations (r = −0.509, p = 0.075) and better right hand visuomotor accuracy on the tracking task (r = −0.509, p = 0.075). A description of the tracking task can be found in Additional file 1.

### Linear regression models

The differentiating regional powers were entered in linear regression analysis for each group separately, and for the composite group of CT + CRT patients and controls. Of the three pursuit variables, visuomotor accuracy of the preferred hand (Dr_pu) is best explained by regional θ power. Within the CT + CRT group, 39.6% variance on Dr_pu could be explained by increased RC θ power. No significant model could be reached for controls.

Attentional fluctuations (SD_sa) could be significantly explained by α2 powers in one or two regions, regardless of group composition. More variance could be explained in the CT + CRT group (R^2^ = 0.653), than in controls (R^2^ = 0.345). D_pu and Dr_pu could be significantly explained by α2 powers in multiple regions. Similarly, more variance could be explained in CT + CRT patients (R^2^ = 0.912 (D_pu) and 0.727 (Dr_pu)) than in controls (R^2^ = 0.173 (D_pu) and 0.327 (Dr_pu)). An overview of linear regression outcomes is given in Additional file 6.

## Discussion

The global power spectrum of CRT‐treated patients reflects a slight increase of θ activity and a significant decrease of α2 activity. Patients treated with CT alone display a power spectrum similar to controls, except for a significantly increased level of left frontal α2 power.

### Late effects of cranial irradiation

The increase in θ and decrease in α2 indicate a tendency towards global slowing of brain oscillatory activity, a finding that is consistent with other recent MEG studies in low grade glioma patients and Alzheimer patients [15,16]. Increased θ power is also known as a nonspecific sign of brain pathology [17]. Our results confirm Tucker’s findings from 1989 of increased θ and decreased α power in irradiated patients and are also in line with results from a review by Klimesch, published in 1999 [18,36]. He concluded that α power is lowered and θ power enhanced in subjects with a variety of different neurological disorders. Also in healthy people in the late part of their lifespan this pattern is familiar and is supposed to reflect some degree of normal aging in the form of vascular or fibrillary degeneration [17]. Taken together with the fact that dementia is frequently reported as a late effect of whole brain irradiation and the neuropsychological deficiencies currently present, these findings suggest that the irradiated brain might be aging faster and could be at risk for early‐onset dementia [18,37-40]. Long-term follow‐up of this patient group is therefore increasingly important. Longitudinal research is also recommended.

High percentages of variance of visuomotor accuracy, especially of the right hand, are explained by increased θ and decreased α2 powers in the CT + CRT group. This is a strong indication that these deviant power levels are underlying this particular deficiency. Unexpectedly, decreased regional α2 powers are significantly associated with less attentional fluctuations in both irradiated patients and controls. In general, α is suppressed by stimuli (e.g. light) and mental activities [17]. Rihs et al. (2009) describe how α power increases and decreases co-vary with visual cortex excitability related to anticipatory visual attention processes [41-43]. This illustrates that patterns of α power fluctuations are difficult to decipher and it is almost impossible to interpolate between resting state and attentional task data.

The CT + CRT group demonstrates decreased α2 levels and *worse* attentional fluctuations. Within the right frontal region of irradiated patients, where the α2 power level is decreased, the association with attentional fluctuations is absent, while there is a strong positive association in controls. This suggests that especially the significantly decreased α2 power in the right frontal region underlies the deficiency in sustained attention in the CT + CRT group. Lower α2 is not helping sustained attention here. The α2 associations in the occipital regions are slightly, although not significantly, stronger than in controls, which could hypothetically indicate that the occipital lobes are trying to compensate for decreased α2 power in the right frontal region.

Additionally, recent research on θ activity, e.g. by Voytek et al. (2010) [44], suggests that increased θ power may reflect compensatory activity for a damaged brain region that is being challenged, rather than reflecting pathology. In our study, subtle implications were found for compensatory θ activity in the left occipital lobe. Combined, the increase in left occipital θ power and the decrease in occipital α2 powers both being associated with less attentional fluctuations, suggest that attentional fluctuations in irradiated patients may be partly counterbalanced by compensatory activity in the occipital lobes. One might arguably postulate that the observed trends for elevated occipital α1 power represent nothing more than a side effect of working with relative powers instead of absolute. These slightly increased levels were not associated with any of the cognitive deficiencies in the irradiated patients, although reports have been made about associations between an increase in lower α power and disturbed working memory [15].

### Late effects of chemotherapy

The patient group treated with CT alone showed no signs of early aging. The subtlety of the cognitive deficiencies and limited spectral power changes are consistent with overall findings that the patients treated with CT only do not perform significantly worse than controls. The significant reduction of late effects makes CT a superior alternative to CRT, especially since there was no trade-off in terms of mortality or recurrence [1,2]. Event-free survival at 10 years after treatment with ALL-6 was even 30% better than after treatment with ALL-5 and CNS relapse rates improved from 12.9% to 1.1% [45]. Meanwhile, suspected neurotoxic effects of CT might be masked by compensatory mechanisms. It remains unclear whether the elevated level of left frontal α2 power should be interpreted as compensatory or pathological. If lowered α2 is regarded as compensatory, increased α2 in the CT patients should be regarded as pathological. However, increased α2 power and the mild cognitive deficiencies observed in the CT patients were not associated, which contradicts the pathology hypothesis. In CT patients, α2 power is not correlated with attentional fluctuations, so the absence of attentional fluctuations combined with increased α2 is not necessarily inconsistent with the hypothesis of lower α2 levels being compensatory. Also, the possibility of increased α2 being compensatory in other cognitive domains cannot be ruled out.

The time that passed since end of treatment (20–25 years) and the age of the patients at time of treatment (an age of relatively high plasticity of the brain) could have allowed for alternative brain strategies to develop. For the CT patients, this could have resulted in mostly normal cognitive performance. For the irradiated group, however, neurotoxicity is much more severe. Compensation might exist, yet appears to be inadequate, which emphasizes this group’s need for long-term follow‐up and interventions.

### Methodological considerations

Although there were good reasons for analyzing relative powers instead of absolute, this also creates a limitation. A decrease in one frequency band might cause an increase in another band, although this would not be the observed pattern in absolute measures. On the other hand, consistency with the literature and significant associations with cognitive deficiencies support our findings in the CT + CRT group. Another possible limitation of the study is the relatively small sample size which raises the question whether this set of subjects is representative for the whole population. Confirmation by future studies is needed.

No corrections were applied for multiple comparisons, although commonly the Bonferroni method would be applied in order to lower the chances of false discovery of significant results (the type I error rate). In a publication by Perneger et al. (1998), a view widely held by epidemiologists is expressed that this method creates more problems than it solves and the best way of dealing with multiple comparisons is by simply describing what tests of significance have been performed, and why [46]. This way, findings are not interpreted differently according to how many other tests were performed. Also, type I errors cannot be prevented without increasing the number of type II errors, which are equally worth avoiding. In the present study, pre-established hypotheses were tested in our correlation analyses, instead of just correlating everything, and more than 50% of these hypotheses were confirmed. In addition, the correlation results were supported by the regression results. This, combined with reporting effect sizes in addition to significance levels, should provide the reader with sufficient information to base an interpretation of the results on.

## Conclusions

Power spectrum analysis of MEG registrations is able to demonstrate abnormal patterns of resting-state oscillatory brain activity in irradiated long-term survivors of ALL. The suspicion of a faster aging brain after CRT warrants careful long-term follow‐up and screening for early‐onset dementia. In future ALL therapy, CRT should be completely abolished.

## Abbreviations

ALL: Acute lymphoblastic leukemia; CT: Chemotherapy; CRT: Cranial radiation therapy; MEG: Magnetoencephalography; ANT: Amsterdam Neuropsychological Tasks program; CNS: Central nervous system; EF: Executive functioning; DCLSG: Dutch Childhood Leukemia Study Group; MTX: Methotrexate.

## Competing interests

The company Sonares BV commercially distributes the ANT program that has been used in this study. Dr. LMJ de Sonneville is director of this firm. The other authors have nothing to declare.

## Authors’ contributions

All authors participated in the discussion and design of the study. LS, AV and CB applied for the grant. Data collection and analysis was done by MD, IS en BD. MD and IS wrote the manuscript. All authors read and approved the final manuscript.

## Funding

This study was supported by a grant to LS, AV and CB from the Dutch Cancer Society (UL 2006–3630).

## Pre-publication history

The pre-publication history for this paper can be accessed here:

http://www.biomedcentral.com/1471-2377/12/84/prepub

## Supplementary Material

Additional file 1 ANTprogram. Description of subtests of the Amsterdam Neuropsychological Tasks program (ANT). Click here for file

Additional file 2 **Global relative powers.** Mean global relative powers per group. Four visualisations of the same data. A: Bar chart of the raw power values, equal to Figure 2 in the main text. B: Bar chart of the log transformed power values. C: Line chart of the raw power values. D: Line chart of the log transformed power values. Error bars represent one standard deviation. Click here for file

Additional file 3 **Regional relative power differences.** Statistics of the multivariate tests and group contrasts of the regional relative powers in each frequency band. (MANOVA 1: theta, MANOVA 2: alpha 1, MANOVA 3: alpha 2, MANOVA 4: beta, MANOVA 5: gamma). Group differences were tested with age as a covariate. Click here for file

Additional file 4 **Neuropsychological outcome.** Overview of group differences on all neuropsychological variables assessed with the Amsterdam Neuropsychological Tasks (ANT) program. Separate ANOVA’s were applied per variable, with simple contrasts between each patient group and controls. Click here for file

Additional file 5 Correlations. Overview of differences between correlation coefficients of regional power and ANT variables, calculated separately within the CT + CRT group and in controls. Click here for file

Additional file 6 **Linear regression models.** Overview of linear regression models within the separate (composite) groups. R^2^ indicates the explained variance of ANT variables by regional powers; p is the significance of each linear regression model. Click here for file
