# Supplementary material for: Long-term effects of cranial irradiation and intrathecal chemotherapy in treatment of childhood leukemia: a MEG study of power spectrum and correlated cognitive dysfunction
Source: BMC Neurol. 2012 Aug 28;12:84. doi: 10.1186/1471-2377-12-84 (PMC3517522; doi:10.1186/1471-2377-12-84)
Supplement: Additional file 1 — ANTprogram. Description of subtests of the Amsterdam Neuropsychological Tasks program (ANT). [file 1471-2377-12-84-S1.pdf]

Additional file 1 – ANTprogram

Description of subtests of the Amsterdam Neuropsychological Tasks program (ANT)

Baseline speed

A simple reaction time task in which cognitive demands are restricted to the mere detection of a stimulus. Provides a reference level for response speed.

Parameters: **T_bs** (mean response time of left and right hand), **S_bs** (standard deviation of response times).

Feature identification

To test speed and accuracy of processing complex abstract visuo-spatial patterns. Task demands include maintenance and manipulation of working memory representations.

Parameters**: Ts_fi** (mean response time in *similar* condition, where target is surrounded by similar patterns and distinction is made upon detailed information processing), **Td_fi** (mean response time in *dissimilar* condition, where target is surrounded by dissimilar patterns and distinction is based on more global and simple information processing), **PES_fi** (percentage of errors in similar condition), **PED_fi** (percentage of errors in dissimilar condition).

Memory search objects

Subjects have to detect a predefined target set in a signal of four 2-dimensional symbols (red, green, blue, yellow / circle, triangle, cross, square). Memory load is increased across two task parts. Part 2 requires continuous monitoring and updating of the contents of the working memory.

Parameters: **T1_2d** = (th1_2d + tc1_2d)/2 (mean of response times *hits* and correct *rejections* in part 1), **T2_2d** = (th2_2d + tc2_2d)/2 (mean of response times *hits* and *correct rejections* in part 2), **Ne1_2d** (mean number of errors part 1), **Ne2_2d** (mean number of errors part 2).

Sustained attention

To evaluate changes and fluctuations in speed and accuracy of processing over time. The paradigm induces a response bias providing indices for response inhibition and behavioral adaptation to feedback.

Parameters: **T_sad** (mean response time), **S_sad** (standard deviation of response time, a measure for fluctuations of attention), **PM_sad** (percentage of *misses;* answer is *no* while it should be *yes*), **PF_sad** (percentage of *false alarms*; answer is *yes* while it should be *no*).

Shifting attentional set

To evaluate inhibition of prepotent responses and attentional flexibility. In the signal - a horizontal bar - a colored square may jump from left to right, or vice versa. Depending on the color of the square, the subject should execute a compatible response (part 1), i.e. press right (left) key when square jumped to the right (left), or is required to execute an incompatible response (press opposite keys, part 2), In part 3, trials of part 1 and 2 are randomly mixed which requires a switch between the two types of response sets.

Parameters: **T_inhib** (mean response time *inhibition*: difference between condition 2 and 1), **T_flex** (mean response time *flexibility*: difference between condition 3 and 1), **P_inhib** (percentage of errors on *inhibition*), **P_flex** (percentage of errors on *flexibility*).

Pursuit

This task evaluates the quality of visuomotor control. The subject has to track a small star, which moves continuously in a random direction across the screen. The task requires concurrent planning and execution of unpredictable movements.

Parameters: **D_pu** (mean distance to target), **S_pu** (standard deviation of D_pu)

Tracking

This task serves the same purpose as the pursuit task, but now the subject is asked to execute planned, more automated movements. The subject has to draw a circle by moving the mouse cursor in-between two large concentric circles on the screen. This task requires less controlled processing than the Pursuit task.

Parameters: **Da_tr** (mean absolute distance to target), **S_tr** (standard deviation of Da_tr).

Diff_D_pu_tr (difference of D_pu and Da_tr) and **Diff_S_pu_tr** (difference of S_pu and S_tr) are measures of working memory.

Visuospatial Sequencing

To evaluate memory of visuospatial temporal patterns. In each trial, a number of circles are pointed out in an array of nine circles, arranged in a 3x3 matrix on the computer screen. The

subject has to point out the same circles in the same order by moving the mouse cursor and press a button when it is positioned at the right location(s). The test consists of 24 trials in which the number of target circles varies from 3 to 7 and in which the spatial sequential patterns increase gradually in complexity.

Parameters: **Nit_vs** (number of correctly identified circles), **Nitco_vs** (number of correctly identified circles in the correct order)

Diff_vs (Nit_vs – Nitco_vs) (or **ratio_vs** (Nit_vs / Nitco_vs)) is a measure of the sequential working memory component.
