# Supplementary material for: Long-term effects of cranial irradiation and intrathecal chemotherapy in treatment of childhood leukemia: a MEG study of power spectrum and correlated cognitive dysfunction
Source: BMC Neurol. 2012 Aug 28;12:84. doi: 10.1186/1471-2377-12-84 (PMC3517522; doi:10.1186/1471-2377-12-84)
Supplement: Additional file 2 — Global relative powers. Mean global relative powers per group. Four visualisations of the same data. A: Bar chart of the raw power values, equal to Figure 2 in the main text. B: Bar chart of the log transformed power values. C: Line chart of the raw power values. D: Line chart of the log transformed power values. Error bars represent one standard deviation. [file 1471-2377-12-84-S2.pdf]

Additional file 2 - Global relative powers

Mean global relative powers per group. Four visualisations of the same data.
A: Bar chart of the raw power values, *equal to Figure 2 in the main text*. **B**: Bar chart of the log transformed power values. **C**: Line chart of the raw power values. **D**: Line chart of the log transformed power values. Error bars represent one standard deviation.

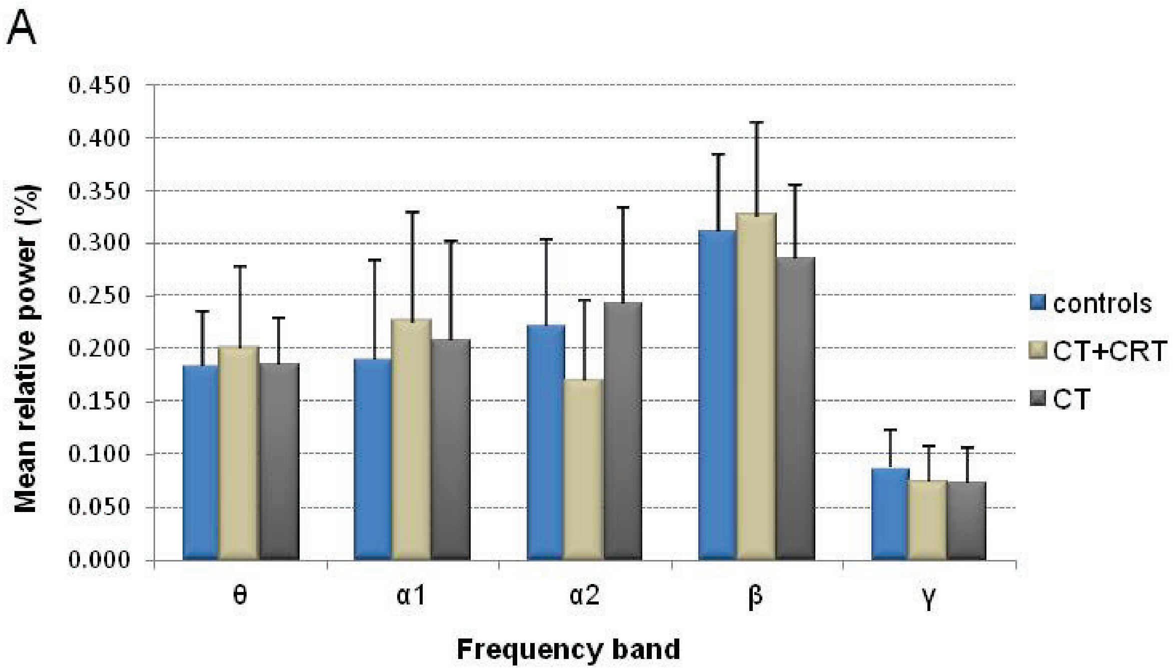

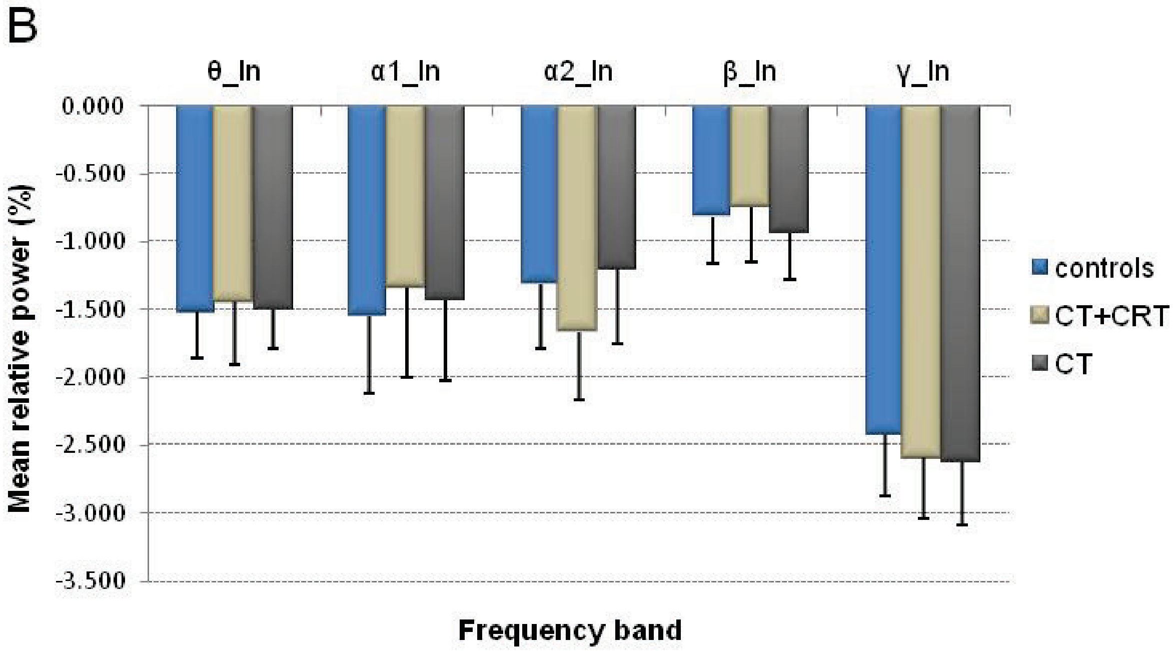

C

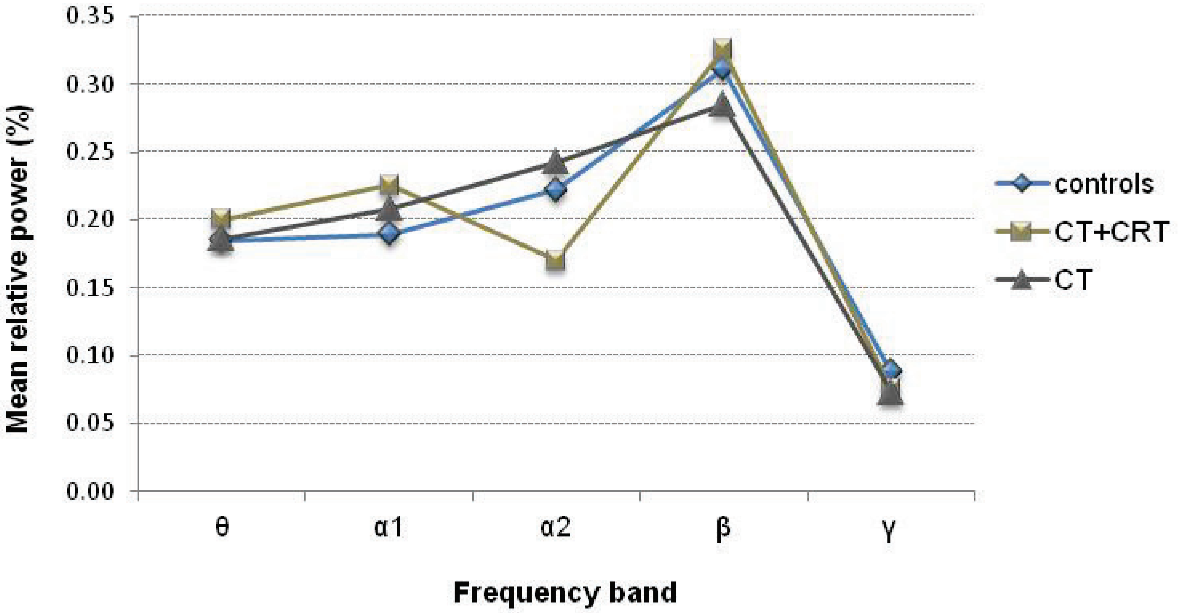

0.35
0.30
0.25
0.20
0.15
0.10
0.05
0.00
Mean relative power (%)
θ
α1
α2
β
γ
Frequency band
controls
CT+CRT
CT

D

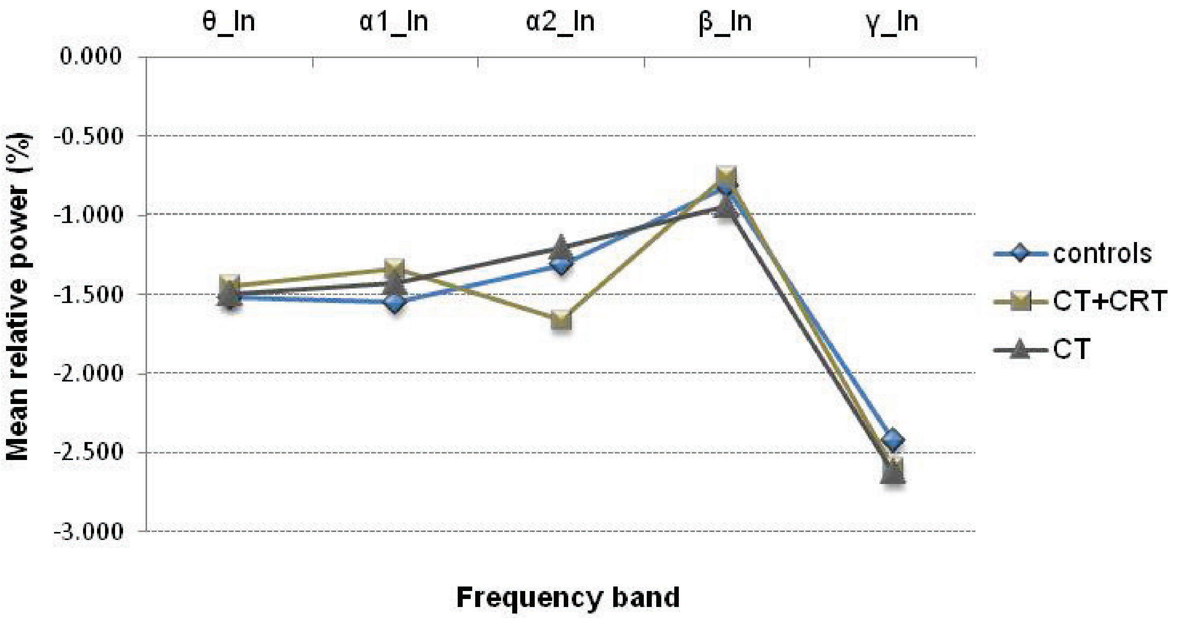

θ_ln
α1_ln
α2_ln
β_ln
γ_ln
0.000
-0.500
-1.000
-1.500
-2.000
-2.500
-3.000
Mean relative power (%)
Frequency band
controls
CT+CRT
CT
