# Supplementary material for: Long-term effects of cranial irradiation and intrathecal chemotherapy in treatment of childhood leukemia: a MEG study of power spectrum and correlated cognitive dysfunction
Source: BMC Neurol. 2012 Aug 28;12:84. doi: 10.1186/1471-2377-12-84 (PMC3517522; doi:10.1186/1471-2377-12-84)
Supplement: Additional file 3 — Regional relative power differences. Statistics of the multivariate tests and group contrasts of the regional relative powers in each frequency band. (MANOVA 1: theta, MANOVA 2: alpha 1, MANOVA 3: alpha 2, MANOVA 4: beta, MANOVA 5: gamma). Group differences were tested with age as a covariate. [file 1471-2377-12-84-S3.pdf]

Additional file 3 - Regional relative power differences

Statistics of the multivariate tests and group contrasts of the regional relative powers in each frequency band (MANOVA 1: theta, MANOVA 2: alpha 1, MANOVA 3: alpha 2, MANOVA 4: beta, MANOVA 5: gamma). Group differences were tested with age as a covariate.

* = statistical trend, ** = significant difference

MANOVA 1	Between-Subjects Effects						Simple contrasts (versus controls)			
θ	**AGE**	Sign. (p)	Effect size (η_p^2)	**GROUP**	Sign. (p)	Effect size (η_p^2)	**CT+CRT group**		**CT group**	
							Sign. (p)	Cohen's d	Sign. (p)	Cohen's d
**Multivariate tests**	F[10,54] = 2.191	0.020	0.306	F[20,108] = 1.251	0.212	0.191	N/A	N/A	N/A	N/A
LC							0.208	0.112	0.381	-0.093
LF							0.284	0.023	0.752	0.102
LO							**0.066***	0.202	0.670	0.355
LP							0.137	0.210	0.793	0.079
LT							0.197	0.010	0.180	-0.150
RC							**0.038****	0.343	0.645	0.030
RF							0.124	0.155	0.950	0.220
RO							**0.051***	0.303	0.644	0.328
RP							**0.029****	0.400	0.506	-0.037
RT							0.147	0.107	0.330	-0.085

MANOVA 2	Between-Subjects Effects						Simple contrasts (versus controls)			
							CT+CRT group		CT group	
**α1**	**AGE**	Sign. (p)	Effect size (η_p^2)	**GROUP**	Sign. (p)	Effect size (η_p^2)	Sign. (p)	Cohen's d	Sign. (p)	Cohen's d
**Multivariate tests**	F[10,54] = 1.712	0.102	0.241	F[20,108] = 0.859	0.638	0.137	N/A	N/A	N/A	N/A
LC							0.105	0.359	0.612	0.254
LF							0.305	0.282	0.738	0.137
LO							**0.081***	0.396	0.664	0.236
LP							0.109	0.351	0.491	0.310
LT							0.202	0.346	0.712	0.158
RC							0.163	0.249	0.625	0.265
RF							0.438	0.177	0.647	0.186
RO							**0.059***	0.462	0.581	0.262
RP							0.153	0.250	0.719	0.236
RT							0.285	0.261	0.945	0.074

MANOVA 3	**Between-Subjects Effects**						**Simple contrasts (versus controls)**			
**α2**	**AGE**	Sign. (p)	Effect size (η_p^2)	**GROUP**	Sign. (p)	Effect size (η_p^2)	**CT+CRT group**		**CT group**	
							Sign. (p)	Cohen's d	Sign. (p)	Cohen's d
**Multivariate tests**	F[10,54] = 1.469	0.177	0.214	F[20,108] = 1.596	**0.067**	0.228	N/A	N/A	N/A	N/A
LC							**0.063***	-0.657	0.192	0.332
LF							0.143	-0.361	**0.030****	0.562
LO							**0.005****	-0.611	0.669	-0.052
LP							**0.038****	-0.576	0.552	0.094
LT							**0.008****	-0.613	0.191	0.221
RC							**0.015****	-0.840	0.134	0.371
RF							**0.034****	-0.590	0.107	0.353
RO							**0.002****	-0.751	0.837	-0.096
RP							**0.018****	-0.691	0.223	0.276
RT							**0.005****	-0.660	0.249	0.188

MANOVA 4	**Between-Subjects Effects**						**Simple contrasts (versus controls)**			
							**CT+CRT group**		**CT group**	
**β**	**AGE**	Sign. (p)	Effect size (η_p^2)	**GROUP**	Sign. (p)	Effect size (η_p^2)	Sign. (p)	Cohen's d	Sign. (p)	Cohen's d
**Multivariate tests**	F[10,54] = 1.033	0.429	0.161	F[20,108] = 0.650	0.865	0.107	N/A	N/A	N/A	N/A
LC							0.704	0.093	0.589	-0.282
LF							0.866	0.190	0.464	-0.319
LO							0.930	0.030	0.372	-0.330
LP							0.790	0.081	0.432	-0.337
LT							0.537	0.265	0.325	-0.342
RC							0.896	0.135	0.326	-0.401
RF							0.605	0.250	0.260	-0.414
RO							0.877	0.102	0.567	-0.224
RP							0.945	0.168	0.399	-0.362
RT							0.647	0.197	0.429	-0.286

MANOVA 5	Between-Subjects Effects						Simple contrasts (versus controls)			
γ	**AGE**	Sign. (p)	Effect size (η_p^2)	**GROUP**	Sign. (p)	Effect size (η_p^2)	**CT+CRT group**		**CT group**	
							Sign. (p)	Cohen's d	Sign. (p)	Cohen's d
**Multivariate tests**	F[10,54] = 1.398	0.206	0.206	F[20,108] = 0.665	0.852	0.110	N/A	N/A	N/A	N/A
LC							0.163	-0.251	0.312	-0.432
LF							0.105	-0.374	0.385	-0.356
LO							0.421	-0.313	0.547	-0.161
LP							0.185	-0.260	0.218	-0.477
LT							0.283	-0.401	0.433	-0.223
RC							0.209	-0.212	0.211	-0.489
RF							0.170	-0.281	0.237	-0.450
RO							0.267	-0.441	0.290	-0.287
RP							0.304	-0.205	0.213	-0.452
RT							0.108	-0.582	0.254	-0.331
