# Supplementary material for: Long-term effects of cranial irradiation and intrathecal chemotherapy in treatment of childhood leukemia: a MEG study of power spectrum and correlated cognitive dysfunction
Source: BMC Neurol. 2012 Aug 28;12:84. doi: 10.1186/1471-2377-12-84 (PMC3517522; doi:10.1186/1471-2377-12-84)
Supplement: Additional file 4 — Neuropsychological outcome. Overview of group differences on all neuropsychological variables assessed with the Amsterdam Neuropsychological Tasks (ANT) program. Separate ANOVA’s were applied per variable, with simple contrasts between each patient group and controls. [file 1471-2377-12-84-S4.pdf]

Additional file 4 – Neuropsychological outcome

Overview of group differences on all neuropsychological variables assessed with the Amsterdam Neuropsychological Tasks (ANT) program. Separate ANOVA's were applied per variable, with simple contrasts between each patient group and controls.
* = statistical trend, **= significant difference

				Simple contrasts versus controls			
**ANOVA**				**CT+CRT group**		**CT group**	
**ANT variable**	**Group**	**Sign. (p)**	**Effect size (η_p^2)**	**Sign. (p)**	**Cohen's d**	**Sign. (p)**	**Cohen's d**
T_bs	F[2,64] = 3.191	**0.048****	0.091	0.120	-0.462	0.171	0.435
S_bs	F[2,64] = 0.504	0.607	0.015	0.785	-0.076	0.408	0.248
Ts_fi	F[2,64] = 1.994	0.145	0.059	0.118	-0.479	0.511	0.203
Td_fi	F[2,64] = 1.123	0.332	0.034	0.254	-0.363	0.583	0.165
diffT_fi	F[2,64] = 2.032	0.139	0.060	0.110	-0.470	0.529	0.201
Pes_fi	F[2,64] = 0.899	0.412	0.027	0.264	-0.303	0.736	0.117
Ped_fi	F[2,64] = 1.133	0.328	0.034	0.185	-0.367	0.816	0.079
T1_2d	F[2,64] = 0.409	0.666	0.013	0.515	-0.195	0.698	0.119
T2_2d	F[2,64] = 1.836	0.168	0.054	0.107	-0.511	0.662	0.130
diffT_2d	F[2,64] = 1.782	0.177	0.053	0.108	-0.518	0.692	0.116
Pe1_2d	F[2,64] = 0.230	0.795	0.007	0.971	-0.009	0.529	0.229
Pe2_2d	F[2,64] = 1.206	0.306	0.036	0.131	-0.439	0.462	-0.229
diffPe_2d	F[2,64] = 1.240	0.296	0.037	0.138	-0.409	0.363	-0.299
S1_2d	F[2,64] = 0.116	0.890	0.004	0.676	-0.124	0.924	0.029
S2_2d	F[2,64] = 0.734	0.484	0.022	0.245	-0.393	0.940	-0.021
T_sa	F[2,64] = 1.231	0.299	0.037	0.125	-0.466	0.774	-0.086
SD_sa	F[2,64] = 1.816	0.171	0.054	**0.062***	-0.523	0.451	-0.248
PM_sa	F[2,64] = 0.143	0.867	0.004	0.817	0.065	0.598	0.170
PF_sa	F[2,64] = 0.003	0.997	0.000	0.987	0.006	0.951	-0.015
T_inhib	F[2,64] = 0.543	0.584	0.017	0.502	-0.203	0.338	-0.289
P_inhib	F[2,64] = 1.854	0.165	0.055	0.552	-0.198	**0.059***	-0.529
T_flex	F[2,64] = 0.563	0.572	0.017	0.338	-0.327	0.909	0.032
P_flex	F[2,64] = 5.089	**0.009****	0.137	**0.002****	-0.750	0.276	-0.471
Da_tr	F[2,64] = 0.094	0.911	0.003	0.960	-0.017	0.696	0.108
S_tr	F[2,64] = 0.574	0.566	0.018	0.915	-0.032	0.331	0.295
Dal_tr	F[2,64] = 0.452	0.638	0.014	0.386	0.317	0.529	0.169
Dar_tr	F[2,64] = 1.138	0.327	0.034	0.143	-0.465	0.849	-0.056
D_pu	F[2,64] = 2.166	0.123	0.063	**0.049****	-0.568	0.253	-0.361
S_pu	F[2,64] = 1.831	0.168	0.054	**0.075***	-0.497	0.972	0.013
Dl_pu	F[2,64] = 1.062	0.352	0.032	0.201	-0.398	0.300	-0.306
Sl_pu	F[2,64] = 0.366	0.695	0.011	0.399	-0.259	0.717	-0.110
Dr_pu	F[2,64] = 3.171	**0.049****	0.090	**0.015****	-0.647	0.286	-0.375
Sr_pu	F[2,64] = 3.306	**0.043****	0.094	**0.029****	-0.511	0.613	0.248
Nit_vs	F[2,64] = 2.394	**0.099***	0.070	**0.065***	0.824	0.103	0.396
Nitco_vs	F[2,64] = 3.247	**0.045****	0.092	**0.023****	0.776	**0.092***	0.469
diff_Nit_Nitco	F[2,64] = 2.639	**0.079***	0.076	**0.036****	-0.700	0.153	-0.404
