# Supplementary material for: Long-term effects of cranial irradiation and intrathecal chemotherapy in treatment of childhood leukemia: a MEG study of power spectrum and correlated cognitive dysfunction
Source: BMC Neurol. 2012 Aug 28;12:84. doi: 10.1186/1471-2377-12-84 (PMC3517522; doi:10.1186/1471-2377-12-84)
Supplement: Additional file 5 — Correlations. Overview of differences between correlation coefficients of regional power and ANT variables, calculated separately within the CT + CRT group and in controls. [file 1471-2377-12-84-S5.pdf]

Additional file 5 – Correlations

Overview of differences between correlation coefficients of regional power and ANT variables, calculated separately within the CT+CRT group and in controls

$^{+}$ = statistical trend, $^{++}$= significant correlation

$$z' = \frac{1}{2}\,{}^{e}\log\left(\frac{1+r1}{1-r1}\right) \qquad z* = \frac{z1' - z2'}{\sqrt{\frac{1}{n1-3}+\frac{1}{n2-3}}}$$

Correlations with theta	Pearson's r_1 (CT+CRT)	Pearson's r_2 (CON)	z*	Significance of difference r_1-r_2
D_pu & θ_RC_ln	0.428	0.052	1.160	N/A
D_pu & θ_RP_ln	0.387	0.151	0.733	N/A
Dr_pu & θ_RC_ln	0.630^{++}	0.100	1.834	$0.01 < \alpha < 0.05$
Dr_pu & θ_RP_ln	0.599^{++}	0.136	1.587	$0.05 < \alpha < 0.10$
Sr_pu & θ_RC_ln	0.405	0.161	0.765	N/A
Sr_pu & θ_RP_ln	0.365	0.156	0.645	N/A

Correlations with alpha2	Pearson's r_1 (CT+CRT)	Pearson's r_2 (CON)	z*	Significance of difference r_1-r_2
SD_sa & α2_LO_ln	0.608^{++}	0.457^{++}	0.607	not significant
SD_sa & α2_LP_ln	0.393	0.466^{++}	-0.256	not significant
SD_sa & α2_LT_ln	0.551^{+}	0.452^{++}	0.379	not significant
SD_sa & α2_RC_ln	0.177	0.490^{++}	-1.022	not significant
SD_sa & α2_RF_ln	0.078	0.549^{++}	-1.542	$0,05 < \alpha < 0,10$
SD_sa & α2_RO_ln	0.666^{++}	0.463^{++}	0.865	not significant
SD_sa & α2_RP_ln	0.372	0.480^{++}	-0.378	not significant
SD_sa & α2_RT_ln	0.598^{++}	0.454^{++}	0.573	not significant
D_pu & α2_LO_ln	-0.551^{+}	-0.046	-1.642	$0,05 < \alpha < 0,10$
D_pu & α2_LP_ln	-0.561^{++}	-0.205	-1.220	not significant
D_pu & α2_LT_ln	-0.706^{++}	-0.024	-2.447	$0,005< \alpha < 0,01$
D_pu & α2_RC_ln	-0.619^{++}	-0.286	-1.228	not significant
D_pu & α2_RF_ln	-0.460	-0.147	N/A	N/A
D_pu & α2_RO_ln	-0.513^{+}	-0.110	-1.306	$0,05 < \alpha < 0,10$
D_pu & α2_RP_ln	-0.504^{+}	-0.294^{+}	-0.720	not significant
D_pu & α2_RT_ln	-0.655^{++}	-0.054	-2.088	$0,005< \alpha < 0,01$
Dr_pu & α2_LO_ln	-0.508^{+}	0.142	-2.011	$0,005< \alpha < 0,01$
Dr_pu & α2_LP_ln	-0.555^{++}	0.007	-1.810	$0,01 < \alpha < 0,05$
Dr_pu & α2_LT_ln	-0.676^{++}	0.114	-2.679	$\alpha < 0,005$
Dr_pu & α2_RC_ln	-0.697^{++}	-0.129	-2.094	$0,005< \alpha < 0,01$
Dr_pu & α2_RF_ln	-0.471	0.001	N/A	N/A
Dr_pu & α2_RO_ln	-0.522^{+}	0.047	-1.791	$0,01 < \alpha < 0,05$
Dr_pu & α2_RP_ln	-0.635^{++}	-0.119	-1.803	$0,01 < \alpha < 0,05$
Dr_pu & α2_RT_ln	-0.613^{++}	0.114	-2.370	$0,005< \alpha < 0,01$
