# Supplementary material for: Long-term effects of cranial irradiation and intrathecal chemotherapy in treatment of childhood leukemia: a MEG study of power spectrum and correlated cognitive dysfunction
Source: BMC Neurol. 2012 Aug 28;12:84. doi: 10.1186/1471-2377-12-84 (PMC3517522; doi:10.1186/1471-2377-12-84)
Supplement: Additional file 6 — Linear regression models. Overview of linear regression models within the separate (composite) groups. R2 indicates the explained variance of ANT variables by regional powers; p is the significance of each linear regression model. [file 1471-2377-12-84-S6.pdf]

Additional file 6 – Linear regression models

Overview of linear regression models within the separate (composite) groups. R^2 indicates the explained variance of ANT variables by regional powers; p is the significance of each linear regression model.

Theta	**CT+CRT & CON**		**CT+CRT**		**CON**	
	**R^2**	**p**	**R^2**	**p**	**R^2**	**p**
**D_pu**	0.087	0.040*	No significant model		No significant model	
factors	*θ_RP*					
**Dr_pu**	0.250	0.001	0.396	0.016	No significant model	
factors	*θ_RC, age*		*θ_RC*			
**Sr_pu**	0.208	0.005	No significant model		No significant model	
factors	*θ_RC, age*					

Alpha2	**CT+CRT & CON**		**CT+CRT**		**CON**	
	**R^2**	**p**	**R^2**	**p**	**R^2**	**p**
**SD_sa**	0.115	0.017	0.653	0.003	0.345	0.001
factors	*α2_RT*		*α2_LP, α2_RO*		*Age, α2_RF*	
**D_pu**	0.188	0.002	0.912	0.008	0.173	0.048
factors	*α2_RC*		*Age, α2_RP, α2_LO, α2_RC, α2_LP, α2_LT, α2_RO*		*α2_RP, α2_LO*	
**Dr_pu**	0.280	0.005	0.727	0.012	0.327	0.034
factors	*α2_RF, α2_LP, α2_LT, α2_RC*		*α2_RP, α2_LP, α2_LT, α2_RO*		*Age, α2_RO, α2_LP, α2_RP, α2_LO*	
